# Effects of age on the soccer-specific cognitive-motor performance of elite young soccer players: Comparison between objective measurements and coaches’ evaluation

**DOI:** 10.1371/journal.pone.0185460

**Published:** 2017-09-27

**Authors:** Halim Hicheur, Alan Chauvin, Steve Chassot, Xavier Chenevière, Wolfgang Taube

**Affiliations:** 1 Sport and Movement Sciences, Dept of Medicine, University of Fribourg, Fribourg, Switzerland; 2 Laboratoire de Psychologie et NeuroCognition, CNRS–UMR 5105, Univ. Grenoble Alpes, Grenoble, France; University of L'Aquila, ITALY

## Abstract

The cognitive-motor performance (CMP), defined here as the capacity to rapidly use sensory information and transfer it into efficient motor output, represents a major contributor to performance in almost all sports, including soccer. Here, we used a high-technology system (COGNIFOOT) which combines a visual environment simulator fully synchronized with a motion capture system. This system allowed us to measure objective real-time CMP parameters (passing accuracy/speed and response times) in a large turf-artificial grass playfield. Forty-six (46) young elite soccer players (including 2 female players) aged between 11 and 16 years who belonged to the same youth soccer academy were tested. Each player had to pass the ball as fast and as accurately as possible towards visual targets projected onto a large screen located 5.32 meters in front of him (a short pass situation). We observed a linear age-related increase in the CMP: the passing accuracy, speed and reactiveness of players improved by 4 centimeters, 2.3 km/h and 30 milliseconds per year of age, respectively. These data were converted into 5 point-scales and compared to the judgement of expert coaches, who also used a 5 point-scale to evaluate the same CMP parameters but based on their experience with the players during games and training. The objectively-measured age-related CMP changes were also observed in expert coaches’ judgments although these were more variable across coaches and age categories. This demonstrates that high-technology systems like COGNIFOOT can be used in complement to traditional approaches of talent identification and to objectively monitor the progress of soccer players throughout a cognitive-motor training cycle.

## Introduction

Physical capacities represent an important component of performance in soccer. However, soccer is also a cognitive activity where players have to select appropriate solutions while facing multiple stimuli (e.g. the ball, moving teammates and opponents, see [1] for a review). The cognitive-motor performance (CMP), defined here as the capacity to rapidly use sensory information and transfer it into efficient motor output, determines performance in almost all sports, including soccer. In fact, most of the game situations in soccer require interactions between the perceptual, memory, decisional and motor systems. These multiple and parallel processing stages begin with the transmission of information from sensory receptors (from visual, proprioceptive/tactile and vestibular sources) to primary sensory areas. The detection-to-perception stage is modulated by the influence of attention and memory on the processing of environmental features, which result in *perceptual decisions* (see [2] and [3] for reviews specific to the visual and somatosensory systems). Perception is thus an active, high-order process where part of the actual sensory flow is compared to memorized ones, resulting in a particular interpretation of the actual situation. The output of this processing can consist in different actions like i) intercepting the ball ii) keeping the ball iii) passing the ball iv) dribbling or v) kicking the ball.

The CMP is often referred to as “perceptual-cognitive” expertise, performance or skill (for a documented review, see [1] and [4]). The integration and consideration of the motor component in CMP’s is motivated by several reasons. First, players with high perceptual skills (e.g. short *perceptual* reaction times) can have poor motor skills: thus, CMP measurements should include both cognitive-motor (e.g. response times) and motor variables (e.g. passing accuracy) to evaluate soccer-specific CMP. This corresponds to the coaches’ emphasis on the need to ‘not only quickly pick up visual information *but* also to use this information to adequately execute the task’. Second, testing situations where players know that they have to respond via a joystick/oral reports can substantially bias their attention towards *external* sources, thereby reducing their personal investment in the task [5]. In contrast, one can reasonably assume that motor responses through real soccer gestures augment the personal investment (attention) of players in the task.

The non-motor component of the CMP was investigated in soccer players using specific and non-specific protocols [1, 4]. *Specific* and *non-specific* refer in these studies to the type of situations being tested. A standard psychological test like the Stroop test [6] is considered as *non-specific* while a task where participants would face a realistic soccer field projected on a screen is considered as *specific*. Compared to age-matched control participants, superior anticipation, decision-making or higher-order cognitive skills were exhibited by soccer players in specific [7, 8] and non-specific [8–11] tasks. These superior perceptual-cognitive abilities (e.g. greater response accuracies and/or shorter reaction times) were found to be associated with specific visual search strategies and verbal reports in expert players. This was observed in a series of tasks replicating realistic situations such as anticipating the future direction of a penalty kick or making the most appropriate choice in simulated defensive/offensive situations [7, 12–15].

Interestingly, these specific strategies implemented by soccer experts are associated with particular brain activity patterns. Indeed, activations of brain areas (known as the Action Observation Network) were found to be stronger in soccer experts compared to controls in a task where participants had to identify deceptive soccer moves [16, 17]. A recent study [18] revealed that repetitive transcranial magnetic stimulation (rTMS) disrupted performance more strongly in soccer experts than novices in a task where participants had to predict the future direction of the ball after having viewed the initial phases of a penalty kick. This was especially evident when rTMS was applied in the dorsal premotor cortex, with a similar tendency observed only for goalkeepers when rTMS was applied over the Superior Temporal Sulcus, a region known to be involved in biological motion perception [19]. This observation and others (e.g. [20]) indicate that perceptual-cognitive skills rely not only on sensory but also on motor cortical areas that are part of the mirror neuron system. How soccer experience progressively generates such changes in the brain activity has not been investigated yet. However, the average hours accumulated per year during childhood (and during adolescence to a lesser extent) in soccer-specific play activity were found to be the strongest predictor of superior anticipation and decision making in soccer players [12].

The aim of this study was twofold. At the theoretical level, we wanted to investigate the respective effects of age and soccer practice experience on the CMP in young elite soccer players in a passing situation, an issue which has received little attention so far. Following earlier reports [12], we assumed that the childhood-to-adolescence transition period represents a critical phase in the development of perceptual-cognitive (and motor) abilities of young soccer players. In particular, we wanted to test the hypothesis that the reactiveness and the passing accuracy of young soccer players improve substantially during the critical pre-adolescence–mid-adolescence period. At the methodological level, we wanted to compare the objectively determined outcome measures of a new high-technology system (COGNIFOOT, patent pending) when assessing the cognitive-motor performance of soccer players in a short pass situation to the more subjective judgements of expert coaches.

## Materials and methods

### Participants

#### Players

Forty-six elite young soccer players, aged between 11 and 16 years, participated in this study. They belonged to the same elite youth soccer academy, which meticulously selects their players. All the selected players trained at the academy during the week and competed (every week-end)–depending on their age–at the elite regional or national levels resulting in a total of 6 (U12 category) to 8.5 (U14 to U16 categories) hours of soccer specific practice per week. They belonged to different age categories: U12 (N = 14, age = 11.56 ± 0.31 years, whole soccer practice experience = 6.57 ± 0.85 years, including 1.21 ± 0.43 years at the elite level), U13 (N = 7, age = 12.59 ± 0.22 years, whole soccer practice experience = 6.71 ± 1.38 years, including 2.71 ± 0.49 years at the elite level), U14 (N = 10, age = 13.56 ± 0.79 years, whole soccer practice experience = 8.00 ± 1.05 years, including 3.50 ± 0.53 years at the elite level; one female player) and U15U16 (U15 category included two extra players from U16, N = 15, age = 14.71 ± 0.43 years, whole soccer practice experience = 9.30 ± 1.07 years, including 3.97 ± 0.61 years at the elite level; one female player). Each category of players was trained by two expert coaches. All participants (coaches, players and players’ parents) provided informed consent and the research procedures were approved by the local ethics committee at the University of Fribourg.

#### Coaches

Nine coaches (three head coaches of the academy, two coaches for each of the U15U16 and U13 categories, and one coach for each of the U12 and U14 categories) participated in the study by providing their judgements about the performance level of the young players under their responsibility (see Procedure section). The coaches were experienced (8.8 ± 6.9 years of coaching practice at the elite level) and certified trainers. They hold the UEFA-Pro (N = 4), UEFA-A (N = 1), UEFA-B (N = 3) or ASF-C (Swiss Federation of Soccer, N = 1, the C diploma corresponds to ground level before the UEFA-B level) licenses.

### Cognifoot system

The COGNIFOOT system (patent pending at the Swiss Federal Institute of Intellectual Property under the reference *CH00215/16*) is a real-time high-technology system combining a visual environment simulator synchronized with motion capture and ball-launching systems. In the present study, we used a first prototype of this system (COGNIFOOT v1—without ball-launching robots) that we installed in a turf-artificial grass playfield on which players could execute real soccer skills while facing a large screen. The whole set-up is detailed below.

#### Playfield and support structures

The playfield size was equal to 8 x 5 x 5 (length x width x height) meters. Artificial-grass floor texture (PurTurf 32, Realsport, Rossens, Switzerland) covered a surface of 8 x 4 meters (length x width). Metallic structures were located around the playfield in order to support motion capture cameras that were placed at a height of 5 meters.

#### Large screen and visual environment projection

**Fig 1:**
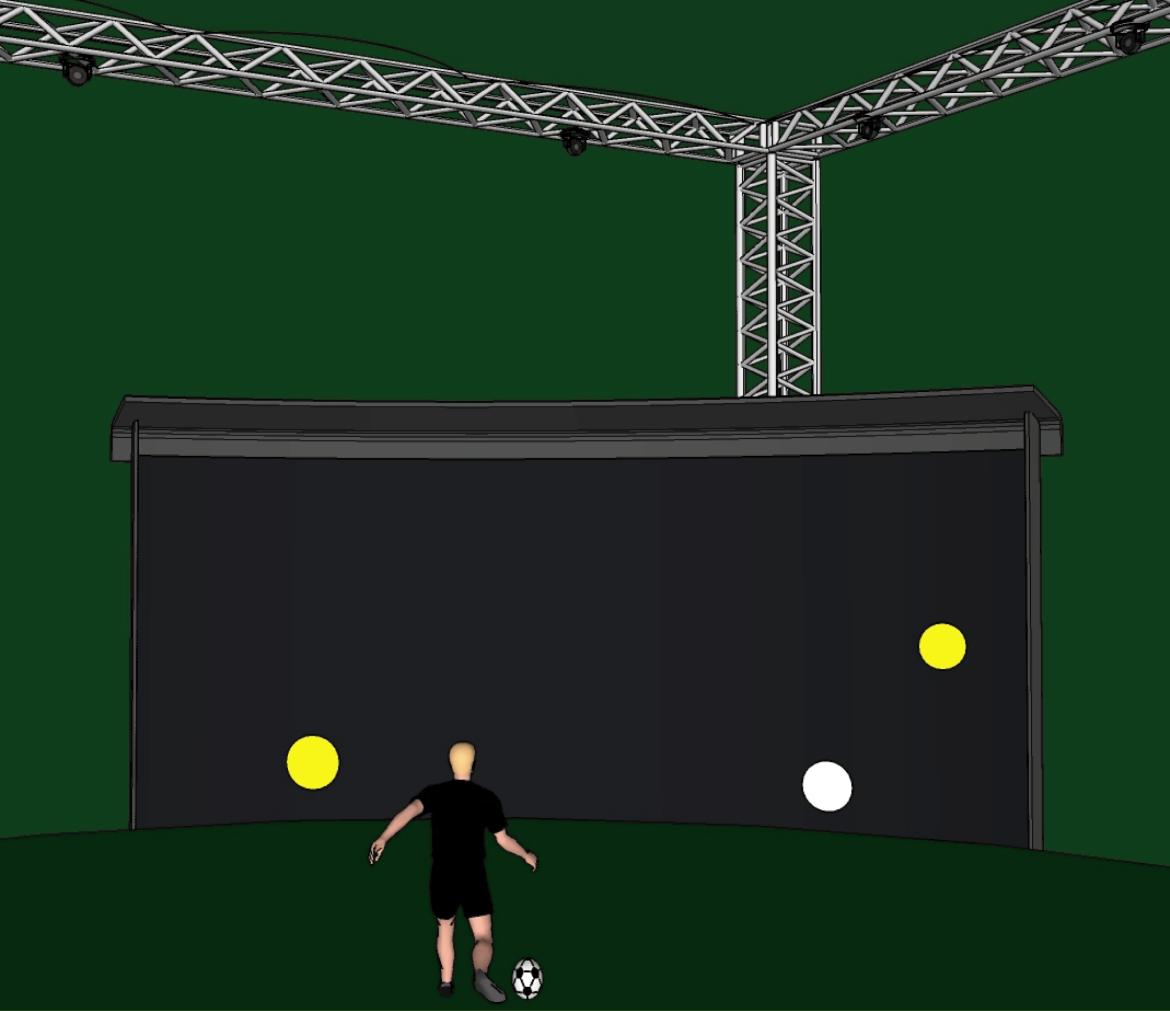
Illustration of the experimental set-up. The player passed the ball towards the white target which could appear at one of 9 randomly-generated positions (here the right-intermediate height position). Visual distractors (yellow circles) could appear randomly on the screen together with the white target. The non-kicking foot had to remain within a black rectangle drawn on the artificial grass. Motion capture cameras were located above the player and tracked the ball movement in real-time. The videoprojector was placed behind the player at a height of 2.72 meters (not shown). Each player performed a total of 108 passes during a test session (see text for details).

A large screen (4 x 3 meters–width x height) made of a shock absorbing tissue was located at a distance of 5.32 meters from the ball position (Fig 1). The visual environment was projected onto the screen using a beamer BenQ MH740 (BenQ corporation Taipei, Taiwan, Full High Definition, luminosity: 4000 ANSI and 3D-compatible) located behind the player at a distance of 6.32 meters to the screen and at a height of 2.72 meters. The beamer was connected to a laptop (HP Elite Book, Hewlett-Packard, Palo Alto, CA, USA equipped with Intel i7 processor, DDR memory: 16 Go) via the HDMI port. The generated image size was equal to 3.80 x 2.01 meters (width x height). The default image background was black (same as the screen color) to ensure a constant contrast of the background between each trial.

### Real-time ball motion tracking and screen calibration

The ball motion was tracked in real-time with 8 Optitrack Prime 17W cameras (NaturalPoint OR, USA). Small infra-red light reflective soft markers were fixed on the ball (standard ball size diameter equal to 22 cm) and after a calibration procedure, the 3D ball position (X, Y and Z spatial coordinates according to a reference frame centered on the initial ball position) was streamed in real-time at a frequency of 360 Hz to the laptop. Ball coordinates were then processed on-line using a self-written “main program” in Matlab (Mathworks Inc., Natick, MA, USA) to compute several parameters related to CMP (see next section). The calibration of the screen was performed using 4 markers located at the corners of the projected image. This allowed a conversion of the target position from pixels to (Y, Z) coordinates (same reference frame used for ball motion tracking). Post-calibration measurements were realized by fixing markers at different locations on the screen (e.g. screen corners and 5 random positions). The spatial coordinates of these markers were then converted into pixel coordinates. Targets of the same size as the one used in the tests were then projected back to the screen using these coordinates. During pilot testing, it was ensured that the distance between each marker and the center of the corresponded (projected) target was systematically below one centimeter. This guaranteed the accuracy of the ball position measurement at impact (whether in pixels or in metric units).

### Reliability of the response time measurements

The accuracy of the automatically computed response times has been verified using a video-based image-by-image control procedure. This has been done manually using video images recorded with a high-speed camera (CASIO EXILIM, Casio Tokyo, Japan—sampling rate: 600 frames per second). The films were taken from a point of view that allowed viewing simultaneously the initial ball position and the screen while the player had to kick the ball towards the target. A fully-identifiable piece of tape was located on the floor at the ball level. The frame by frame scroll of image was performed for 9 consecutive passes. The frames at which the stimulus appeared on the screen and at which the ball went beyond the piece of tape were visually detected. The delay between these instants was converted from a number of frames into time units (ms). The correlation coefficient between these manually-computed response times and automatically-computed response times (COGNIFOOT v1 main program) was equal to 0.99, guaranteeing that our computation of response times was reliable.

### Procedure

#### Visual stimulus

The properties of the visual environment (e.g. the number, location and duration of the stimuli, inter-trial displays) were programmed using self-written Matlab routines and the Psychophysics Toolbox extensions [21–23] in Matlab. A total of 108 stimuli (one stimulus per trial) were used during a single test. The player faced a large screen onto which a white circular target (diameter = 0.20 meter) appeared at one of 9 randomly-generated positions: eccentricities (LEFT, CENTER, RIGHT) x vertical positions (FLOOR, INTERMEDIATE and HIGH). The CENTER position of the target was located directly in front of the player/initial ball position (distance of 5.32 m, 0 degree of visual angle along the eye-target longitudinal axis, perpendicular to the screen). The LEFT and RIGHT positions were located -1.30 and +1.30 meters away (-14.57 and 14.57 degrees of visual angle) from the player. The FLOOR, INTERMEDIATE and HIGH vertical positions were displayed at a height of 0.08, 0.38 and 0.68 meter with respect to the floor level, respectively.

The target appeared alone in one-third of the trials. Yellow circular distractor(s) (same size as the target) appeared together with the target in the rest of the trials. The number of distractors could be equal to 1 or 2 and the distractors’ positions were randomly generated for each trial. The target plus the distractor(s) constituted one stimulus. The duration of appearance of this stimulus was randomly altered between trials to be 200, 300, 400 or 500 ms. Therefore, a total of 9 target positions x 3 distractors’ possibilities (0, 1 or 2) x 4 stimulus durations (200, 300, 400 or 500 ms) resulting in 108 different conditions were tested for each player/test.

### Passing test

Players were required to pass the ball “as accurately and as quickly as possible” towards the target. They were walking in place nearby the ball which was always placed at the same initial position (5.32 m). In order to maintain a good consistency when measuring the response times, players were not allowed to execute more than one step prior to foot-to-ball contact. This adjustment step usually corresponded to re-orienting the non-kicking foot which had to remain within a black rectangle drawn on the floor (Fig 1). Participants could not anticipate the location of the targets as they were displayed at random positions. A trial begun with a sound emitted by the main program and was followed one second later by the visual stimulus onset. The player then executed a pass towards the target. Once the ball had hit the screen, it was sent back to the player by two assistants (usually teammates) who were located at the edges of the screen (Fig 1). The player then placed the ball at the initial position and waited for the next sound. A period of 13 seconds separated the end of the stimulus appearance and the sound announcing the subsequent trial so that a single trial lasted at least 14.2 s. A rest period of 30 seconds was included every 18 trials. A total of 108 passes were performed: this number roughly corresponds to the maximal number of passes for the top 3 central midfielders observed in high-level competition matches [a mean number of passes of 70 ± 30 passes in the study of [24]]. The passing test lasted 30 to 35 minutes.

#### Pre-tests and experimental session recordings

Before performing the actual test, a pre-test of 16 passes was performed by each player to become familiar with the task. The trials were similar to the ones used during the real test except that visuo-auditive feedback indicating pass success or failure was provided after each pass. Players were orally instructed to find the optimal trade-off between reactiveness and passing accuracy. Such feedbacks were not provided during the actual test. A typical experimental session (explanation of the task, pre-test and test) lasted 40 minutes per player. Players usually came by groups of three to serve as “passer” and “ball boys/girls”. The experiments took place in a covered hall within the Realsport Company in Rossens (Switzerland).

#### Ecological considerations

The ecological relevance of the tested task(s), the “stimulus-response” compatibility, the accuracy of the measurements and the discriminative power of the tests and the type (stationary/movement) of response should be taken into account [4, 14] when assessing the CMP in a soccer-specific environment. Another consideration that was directly addressed in the present study is the objectivity of the CMP measurement: passing or choice accuracy was often measured through human observation [4]. In the same vein, the reactiveness of players in a specific passing situation was not accurately measured in all of the above-mentioned studies but one [25]. Here, we took into account each of these considerations although we had to find a trade-off between the practical relevance of the visual-motor conditions and the need to accurately monitor both the properties of the visual environment and the players’ responses. At the visual level, the choice of such simple objects (vs video-clips of real soccer situations used in some of the mentioned studies) was motivated by the observation that simple non-specific visual stimuli (comparable to the ones used here) are sufficient to discriminate between experts and novices’ performances [8, 10]. The choice of different randomly-generated spatial positions of the target and the random appearance of visual distractors was motivated by the need to maintain a high level of attention of the player throughout the test.

At the motor execution level, the vertical positions were chosen to match different heights of passes. Relatively low heights were selected because higher positions would not have been realistic at this particular player-screen distance (5.32 meters). Besides, players often execute passes to teammates while being “pressed” by opponents who can be distinguished solely on the basis of the color of their uniforms: the different stimuli durations we tested and the presence of distractors were dedicated to testing players in close-to-limit visual conditions comparable to the ones encountered in a real game. This experimental design generated different levels of spatial (presence of distractors) and temporal (duration during which information is available to the player) pressures, hence different levels of task difficulty. Taken together, we expected that based on the different difficulty levels, these conditions would be sufficiently demanding to discriminate between different performance levels of players of different ages.

### Measures

#### Passing spatial error

The passing spatial error (PSE) was measured as the distance (in centimeters) between the ball position at impact (on the screen) and the target position on the screen. The ball center and the target center were used to compute this distance.

#### Passing speed

The pass speed (PS) was monitored by dividing the 3D distance traveled by the ball (from its initial position to screen impact position) by the corresponding temporal interval (the delay between the initial ball movement and the ball impact).

#### Response time

The reactiveness of the player was assessed by computing the response time (RT). RT was computed as the delay between the instant of stimulus onset and the first instant of ball motion. The trials where players shot before the appearance of the stimulus (negative response time) were excluded from the analysis. This represented a total of 9 trials observed in 9 different players (out of 972 trials executed by these players).

#### Coaches’ judgments

The coaches were not present during the tests. Before providing them any information about the performance of their players during the *passing* tests, they were asked to assess three aspects of the CMP during a *short-pass situation* based on their experience with the players during games and training. This was done through individual interviews between coaches and the same experimenter. A questionnaire was filled by each coach and the role of the experimenter was to explain the assessment procedure and instructions to the coaches. Each coach (or head coach) was instructed to judge only players that they know. We collected a total of 201 judgments (33, 24, 69 and 75 judgments for the U12, U13, U14 and U15U16 groups, respectively) across all coaches.

Absolute judgments: Each coach had to judge each player using 3 graduated horizontal scales for assessing, from *low* to *high*, the reactiveness (RE), the passing accuracy (PA) and the passing speed (PS) of the players in short pass situations. The scales were 5 centimeters-long, had 0.1 centimeter intervals and were printed on the questionnaire for each player and parameter (0.1 point interval): coaches had to trace a vertical line on the scale to assess the score of a particular player. After each judgment, coaches had to tell the degree of certainty (DC) in their judgments on another scale ranging from complete uncertainty (0%) to complete certainty (100%), using a graduated horizontal placed below each 5-point scale.

Relative judgments: Once they had filled out the whole questionnaire, coaches were asked to repeat the procedure but using relative judgments: here, coaches were told the name of a reference player. This player belonged to the U15U16 group and obtained the best cumulated COGNIFOOT scores and was considered as the best (or one of the three best) players by coaches. Coaches were told that the maximal score of 5 points was the score of the reference player. They then had to provide their judgments relatively to the score of this reference player using the procedures described previously, but with a different color. The objective of this instruction was to force coaches to take into account the actual performance of a player in relation to this reference player and not rate the player solely within his age-group.

#### Coaches’ judgements vs Cognifoot measurements

The COGNIFOOT measurements (PSE, PS and RT) were converted into PA, PS and RE scores using the same 5-point scales, as follows. We first assigned the maximum scores PA_max_, PS_max and_ RE_max_ (5 points) to the (measured) lowest passing spatial error PSE_lowest_, the fastest passing speed PS_fastest_ and the shortest response time RT_best_ measured across all players. The PSE_player_, PS_player_ and RT_player_ were then converted into a 5-point scale by computing the scores PA_score_, PS_score_ and RE_score_ where PA_score_ = (PSE_lowest_ / PSE_player_) / PA_max_, PS_score_ = (PS_player_ / PS_fastest_) / PS_max_ and RE_score_ = (RT_shortest_ / RT_player_) / RE_max_. The PA_scores_, PS_scores,_ and RE_scores_ computed with COGNIFOOT and the ones provided by coaches were then compared.

### Statistical analysis

The mean CMP parameters reflect the global passing performance of a player across all trials and were computed as the mean value (PSE, PS or RT) across all recorded passes of this particular player. The effects of age (continuous predictor) on the mean CMP parameters (dependent variables) were assessed using a linear regression model which included 46 data points (players). The normality of each of the computed distributions was verified using Kolgomorov-Smirnov tests. In a second stage, the same linear regression model was performed with soccer experience as a predictor. This was tested to determine whether the accumulated soccer experience, in addition to age, could also predict the mean CMP. The strength of the correlation was measured using the correlation coefficient *r* and the parameters of the regression line equation (intercept and slope). In particular, the slope was used to measure the age-related and soccer experience-related change in performance over years.

Another linear regression model (with age as a continuous predictor variable) was then used for individual CMP parameters (PSE, PS and RT, dependent variables): this model allowed discriminating between the tested conditions. In particular, we assessed the potential effects of the target position (3 eccentricities x 3 heights), the number of distractors (N = 3), the stimulus duration (N = 4) and the interaction effects on the CMP, in addition to the effect of age. The normality of each of the computed distributions was here also verified using Kolgomorov-Smirnov tests prior to performing linear regressions. A total of 4879 data points was used for this model: 108 stimuli x 46 players = 4968 *minus* 9 incorrect trials *minus* 80 missing passes for one player of the U14 group (for whom we met hardware problems with the laptop after 28 passes–data of this player were not included in the model). These data are detailed in the supplementary material S1. For all regressions, the significance level was fixed to p<0.05. Any significant main and interaction effects were followed up using Bonferroni-corrected pairwise comparisons.

The internal consistency of individual coaches’ scores (e.g. how much coaches changed their judgments across players, irrespective of the inter-coaches difference in the assessment of a particular player) was measured using the Cronbach’s Alpha (α) coefficient [26]. A value of α below 0.7 indicates that the consistency of coaches’ judgment is questionable [26]. Repeated-measures ANOVAs (2 types of judgments x 9 coaches) were performed on DCs to compare the DCs across coaches and type of judgments. Any potential interaction effect (coach x type of judgment) is mentioned in the text.

## Results

### Cognifoot measurements

#### Passing accuracy

**Fig 2:**
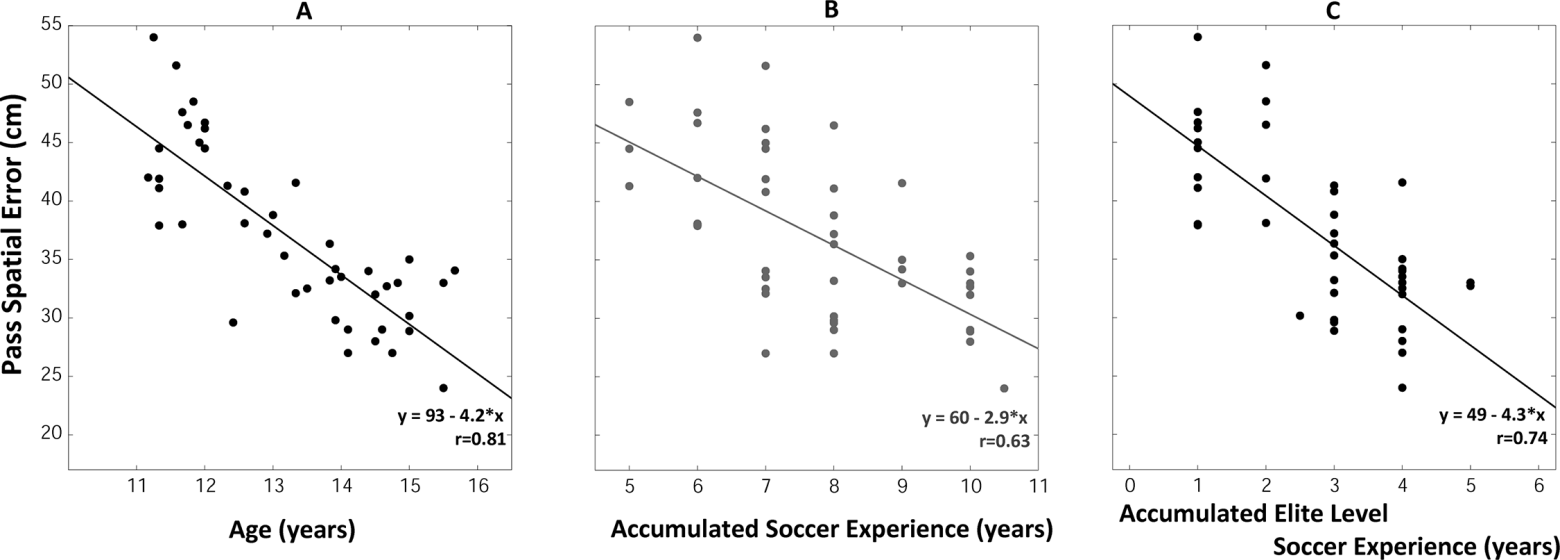
**Passing spatial error of elite young soccer players** as a function of age (A), accumulated soccer experience (B) and elite level-soccer experience (C).

The mean passing spatial error computed across the 108 passes performed by each player is presented in Fig 2. These data followed a linear relationship (F_(1, 44) =_ 84.73, p<0.001, r = 0.81, Fig 2A): the passing spatial error decreased by 4 cm per year of age: it was equal to 45 ± 0.05 cm on average in the youngest (U12 group) players and reached a value of 30 ± 0.03 cm in the U15U16 group. Linear relationships were also observed when plotting the passing spatial error against the accumulated soccer experience (F_(1, 44)_ = 29.77, p<0.001, r = 0.63, Fig 2B) or the accumulated *Elite Level* soccer experience (F_(1, 44)_ = 54.09, p<0.001, r = 0.74, Fig 2C). The regression parameters (e.g. correlation coefficient and slope of the regression line) observed for the *Elite Level* soccer experience (r = 0.74, slope = - 4.3) were comparable to those observed for age (r = 0.81, slope = - 4.2), but differed from those observed for the accumulated soccer experience (r = 0.63, slope = - 2.9, see Fig 2).

The linear regression analysis applied on all recorded passes confirmed the effect of age (F_(1, 43)_ = 88.1, p<0.001). Statistically significant interaction effects were observed (target lateral position and vertical position, the number of visual distractors and the duration of the stimuli—see S1 File for details of all statistical effects, in particular Figure A in S1 File) In particular, passes directed at the floor level were more accurate than passes in other target positions (see supplementary material, page 1).

#### Passing speed

**Fig 3:**
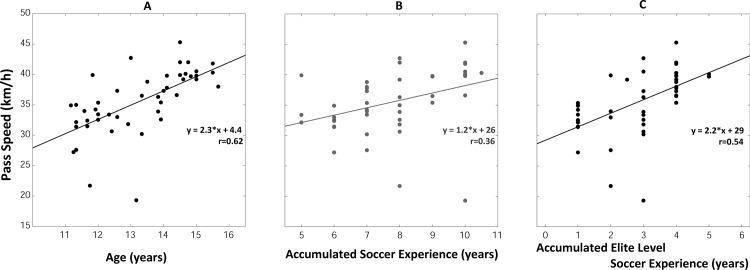
**Passing speed of elite young soccer players** as a function of age (A), accumulated soccer experience (B) and elite level-soccer experience (C).

The mean passing speed as a function of age is presented in Fig 3. A linear relationship was observed (F_(1, 44)_ = 28.19, p<0.001, Fig 3A): the passing speed increased gradually with age. The strength of this relationships (r = 0.62) was affected by the presence of two outliers corresponding to two (U12 and U14) players who kicked the ball at low speeds (around 20 km/h, Fig 3A). Notably, these players did not exhibit such outliers for passing accuracy or response time. The passing speed was equal to 31.7 ± 3.8 km/h on average for the youngest players (U12 group) and increased by 2.3 km/h per year to reach 40.3 ± 2.0 km/h in the U15U16 group. Linear relationships were also observed when considering the accumulated soccer experience (F_(1, 44)_ = 6.68, p = 0.013, r = 0.36, Fig 3B) or the accumulated *Elite Level* soccer experience (F_(1, 44)_ = 17.9, p<0.001, r = 0.54, Fig 3C). As for the passing spatial error/age regressions, regression parameters observed for the *Elite Level* soccer experience (r = 0.54, slope = 2.2) were comparable to those observed for age (r = 0.62, slope = 2.3), but differed from those observed for the accumulated soccer experience (r = 0.36, slope = 1.2, see Fig 3).

The linear regression analysis applied on all recorded passes confirmed the main effect of age (F_(1, 43)_ = 27.6, p<0.001) on the passing speed. However, no statistically significant effect of the target lateral position and vertical position, the number of visual distractors and the duration of the stimuli on the passing speed was observed (p>0.05, no interaction effect–see supplementary material, pages 2–3 and Figure B in S1 File).

#### Response time

**Fig 4:**
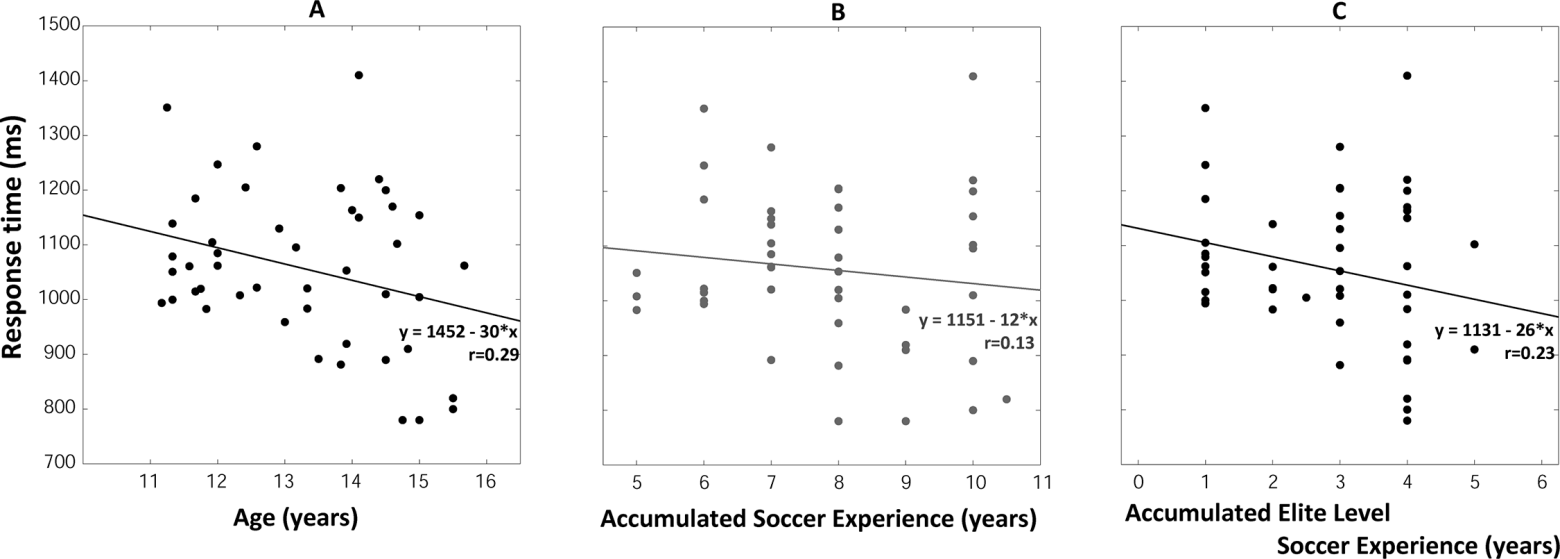
**Response times of elite young soccer players** as a function of age (A), accumulated soccer experience (B) and elite level-soccer experience (C).

The mean response time as a function of age is presented in Fig 4. A linear relationship was observed (F_(1, 44)_ = 4.11, p = 0.048; Fig 4A): response times linearly decreased with age. The mean response time was equal to 1100 ± 102 ms on average for the youngest players (U12 group) and decreased by up to 30 ms per year to reach 1027 ± 193 ms in the U15U16 group. However, the dispersion of the data points for all ages (Fig 4A) was greater than for the passing accuracy (and speed) parameters, resulting in weak correlation coefficient between age and response time (r = 0.29). Such linear relationship was not statistically confirmed for the response time/accumulated soccer experience (p>0.05, r = 0.13, Fig 4B) and the response time/accumulated *Elite Level* soccer experience (p>0.05, r = 0.23, Fig 4C).

The linear regression analysis applied on all recorded passes confirmed the main effect of age on response times although the effect was just at significance level (F_(1, 43)_ = 4.01, p = 0.051). We observed a statistically significant effect of the vertical target position (and of age, see supplementary material): response times were shorter with increasing age and for targets located at floor level (see supplementary material S1, pages 3–4 and Figure C in S1 File).

### Coaches’ judgements vs Cognifoot measurements

In this section, we focus on the scores provided by coaches in order to a) determine the extent to which they were also affected by the age of the players and b) to test how close they were to the objective measurements provided by the COGNIFOOT system.

#### Passing accuracy judgments

**Fig 5:**
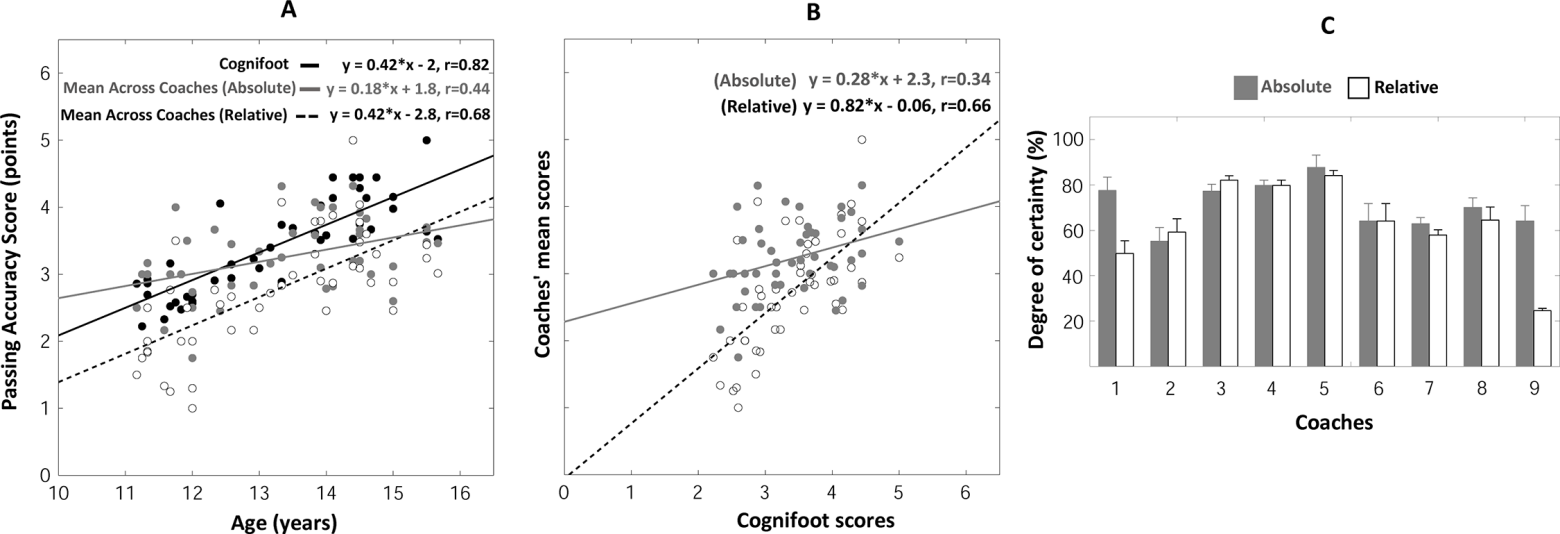
**Passing accuracy scores** as a function of age, measured by the Cognifoot system or judged by coaches (A)—Cognifoot scores vs mean coaches’ scores (B) Degrees of certainty of the coaches in their -passing accuracy- judgments (C).

The PA_score_ measured by the COGNIFOOT system linearly increased with age (F_(1, 44)_ = 91.8, p<0.001, r = 0.82, Fig 5A). The individual *absolute* and *relative* PA_scores_ provided by coaches also linearly increased with age although they were marked by a higher dispersion of data points compared to COGNIFOOT scores, resulting in weaker correlation coefficients (see page 5 and Figure D of S1 File for details). In contrast, the age / mean PA_score_ (Fig 5A) regressions were comparable to those obtained with COGNIFOOT scores (F_(1, 44)_ = 10.7, p<0.01, r = 0.44 and F_(1, 44)_ = 37.7, p<0.001, r = 0.68, respectively for the *absolute* and *relative* judgments, Fig 5A). In particular, the similarity between mean *relative* coaches’ judgments and COGNIFOOT scores was further documented by plotting the coaches’ mean scores *vs* COGNIFOOT scores (Fig 5B). This revealed that mean *relative* PA_scores_ and COGNIFOOT scores were linearly correlated (F_(1, 44)_ = 34.5, p<0.01, r = 0.66).

#### Passing speed judgments

**Fig 6:**
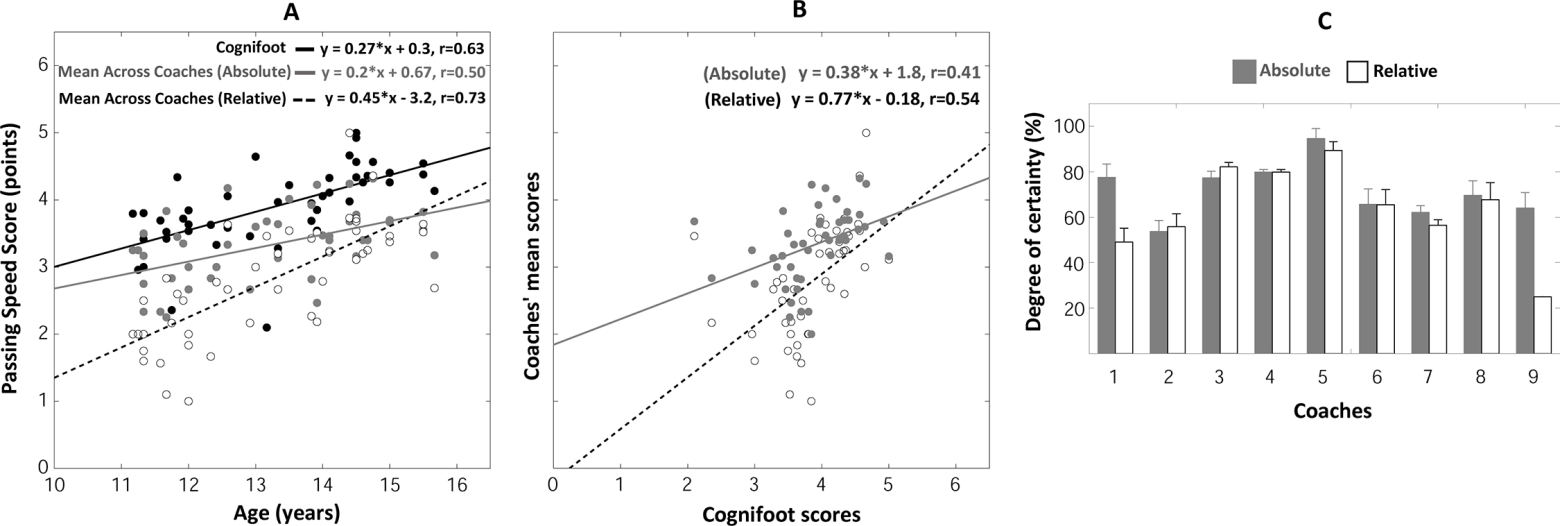
**Passing speed scores** as a function of age, measured by the Cognifoot system or judged by coaches (A)—Cognifoot scores vs mean coaches’ scores (B) Degrees of certainty of the coaches in their -passing accuracy- judgments (C).

The PS_score_ measured by the COGNIFOOT system linearly increased with age (F_(1, 44)_ = 28.6, p<0.001, r = 0.63, Fig 6A). The individual *absolute* and *relative* PS_scores_ provided by coaches also linearly increased with age (see page 6 and Figure E of S1 File for details) although the dispersion of data points of PS_scores_ was higher than the one of COGNIFOOT scores, resulting in weaker correlation coefficients. Computing the mean PS_score_ across coaches (Fig 6A) considerably reduced data dispersion and augmented correlation coefficients (F_(1, 44)_ = 14.4, p<0.001, r = 0.50 and F_(1, 44)_ = 49.7, p<0.001, r = 0.73, respectively for the *absolute* and *relative* judgments, Fig 6A). The similarity between mean *relative* coaches’ judgments and COGNIFOOT scores was further documented by plotting the coaches’ mean scores *vs* COGNIFOOT scores (Fig 6B). This revealed that mean *relative* PS_scores_ and COGNIFOOT scores were linearly correlated (F_(1, 44)_ = 18.2, p<0.001, r = 0.54).

#### Reactiveness judgments

**Fig 7:**
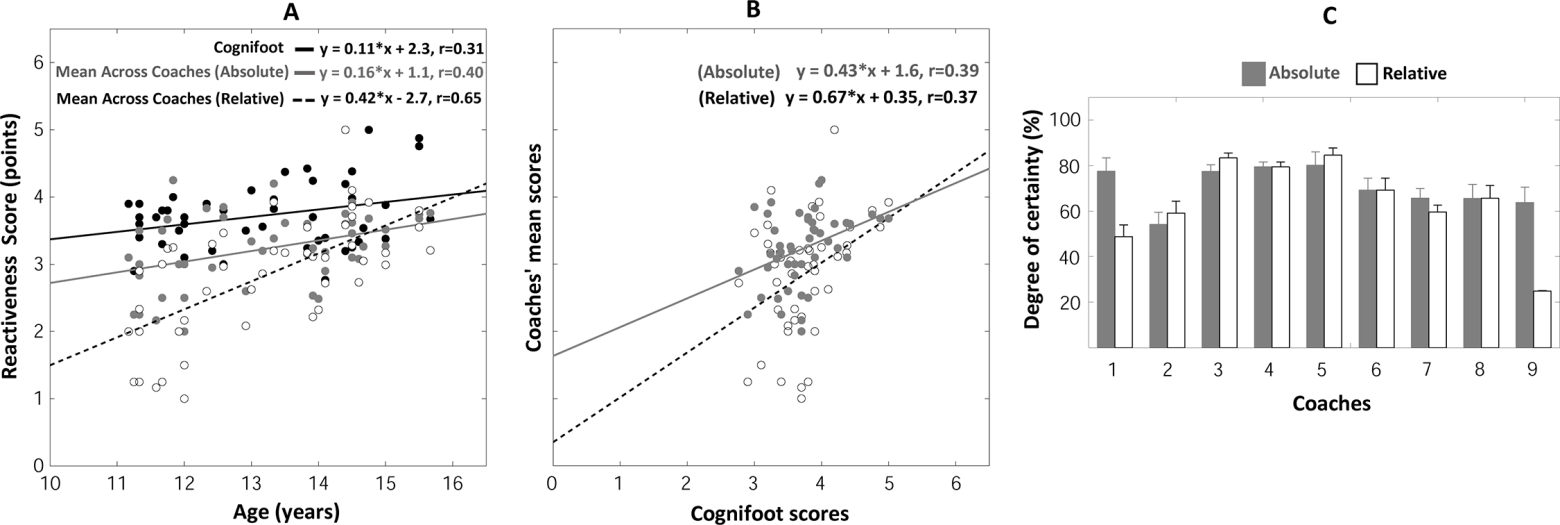
**Reactiveness scores** as a function of age, measured by the Cognifoot system or judged by coaches (A)—Cognifoot scores vs mean coaches’ scores (B) Degrees of certainty of the coaches in their -passing accuracy- judgments (C).

The RE_score_ measured by the COGNIFOOT system linearly increased with age (F_(1, 44)_ = 4.7, r = 0.31, p = 0.036, Fig 7A). The individual *absolute* and *relative* RE_scores_ provided by coaches also linearly increased with age (see pages 6–7 and Figure F of S1 File for details). The mean RE_score_ across coaches (Fig 7A) also linearly increased with age (F_(1, 44)_ = 8.33, p<0.01, r = 0.40 and F_(1, 44)_ = 32.4, p<0.001, r = 0.65, respectively for the *absolute* and *relative* judgments, Fig 7A). Interestingly, the effect of age on the reactiveness of players was stronger in mean *relative* coaches’ data than in COGNIFOOT data (0.65 *vs* 0.31, respectively).

#### Self-estimated reliability of coaches’ judgments

Coaches provided nearly-similar DCs when judging passing accuracy, passing speed or reactiveness of a particular player, resulting in comparable DCs’ values (Figs 5C, 6C and 7C) for these three parameters: DCs typically ranged between 55 and 95% of certainty (except for two *relative* judgments in coaches 1 and 9). Repeated-measures ANOVAs performed on DCs revealed that DCs significantly varied across coaches for passing accuracy (F_(8, 185)_ = 54,9, p<0.001, Fig 5C), passing speed (F_(8, 185)_ = 72,5, p<0.001, Fig 6C) and reactiveness (F_(8, 185)_ = 60,6, p<0.001, Fig 7C). DCs of *absolute* judgments (averaged across coaches) were found to be significantly higher than DCs of *relative* judgments for passing accuracy (F_(1, 185)_ = 100,8, p<0.001, Fig 5C), passing speed (F_(1, 185)_ = 101,3, p<0.001, Fig 6C) and reactiveness (F_(1, 185)_ = 70,9, p<0.001, Fig 7C). A significant (coach x type of judgment) interaction effect was observed for passing accuracy (F_(8,185)_ = 56,1, p<0.001), passing speed (F_(8,185)_ = 53,3, p<0.001) and reactiveness (F_(8, 185)_ = 68,0, p<0.001). However, the difference between *absolute*/*relative* DCs was mainly induced by *relative judgments* data of coaches 1 and 9. No statistically significant difference between *absolute*/*relative* DCs was observed when excluding these coaches for all tested parameters (p>0.05). Notably, only coaches 5 and 6 orally reported that *relative* judgments were easier to provide while coaches 1 and 9 declared that *absolute* judgments were easier to provide.

#### Variability of coaches’ judgments

The Table 1 was designed to complement the observation of a higher dispersion of data points of coaches’ scores (see also supplementary material) compared to COGNIFOOT scores. Here, we isolated the judgments provided by each coach and computed the root mean squared error (RMSE) of the linear regression fit between scores and age performed only for the sample of players judged by each coach. The RMSE indicates how variable the scores around the linear fit were. The corresponding COGNIFOOT scores were extracted for the same samples to allow comparing the variability of coaches and COGNIFOOT scores. For all coaches, the RMSEs values (see Table 1) were systematically higher than the scores obtained with COGNIFOOT. Only coach 3 provided scores with such a low magnitude of variability that was comparable to the COGNIFOOT scores. Averaging coaches’ scores across players (Mean Coaches’ scores, as those shown for the whole population in Figs 5B1, 6B1 and 7B1) resulted in RMSE of similar magnitude between coaches and COGNIFOOT although the variability of COGNIFOOT scores was still systematically lower. These observations further show that individual coaches’ judgments were more variable than COGNIFOOT measurements.

**Table 1:** Root-mean-square-error (RMSE) of the age/scores regressions performed for every sample of players judged by coaches.

Coach	N	Passing Accuracy			Passing Speed			Reactiveness		
		*Abs*	*Rel*	*Cog*	*Abs*	*Rel*	*Cog*	*Abs*	*Rel*	*Cog*
1	44	0,77	0,84	0,40	0,69	0,75	0,48	0,60	0,76	0,47
2	36	0,59	0,66	0,42	0,64	0,60	0,49	0,53	0,65	0,51
3	28	0,49	0,49	0,44	0,50	0,48	0,50	0,49	0,49	0,56
4	24	0,59	0,61	0,44	0,52	0,54	0,48	0,67	0,68	0,57
5	13	0,78	1,01	0,36	0,72	0,97	0,61	0,89	1,01	0,44
6	11	0,51	0,65	0,34	0,47	0,39	0,44	0,86	0,62	0,46
7	14	0,89	1,06	0,28	0,76	0,93	0,43	0,84	1,12	0,31
8	9	0,84	1,01	0,33	0,74	0,54	0,30	0,70	0,63	0,44
9	22	0,65	0,68	0,45	0,62	0,70	0,34	0,76	1,00	0,60
**Mean**	**46**	**0,51**	**0,64**	**0,40**	**0,49**	**0,59**	**0,47**	**0,51**	**0,67**	**0,47**

Abs, Rel and Cognifoot correspond to *absolute*/*relative* coaches’ judgments and COGNIFOOT scores, respectively. The last “virtual” coach (Mean Coach) scores were computed by averaging the scores of different coaches for a same player. Note that except for coach 3, COGNIFOOT’ RMSEs are systematically lower than coaches’ RMSEs, denoting a higher variability in coaches’ scores. Averaging coaches’ scores across players (Mean Coach) resulted in RMSE of similar magnitude between coaches and COGNIFOOT although the variability of COGNIFOOT scores is still systematically lower (see text for details).

#### Internal consistency of coaches’ judgments

The *absolute* coaches’ scores performed for the group A had an internal consistency (α Cronbach coefficient) of α_passing accuracy_ = 0.88, α_passing speed_ = 0.68 and α_reactiveness_ = 0.68. The *relative* coaches’ scores performed for the group A had an internal consistency of α_passing accuracy_ = 0.87, α_passing speed_ = 0.65 and α_reactiveness_ = 0.73. The *absolute* coaches’ scores performed for the group B had an internal consistency of α_passing accuracy_ = 0.83, α_passing speed_ = 0.85 and α_reactiveness_ = 0.85. The *relative* coaches’ scores performed for the group B had an internal consistency of α_passing accuracy_ = 0.87, α_passing speed_ = 0.70 and α_reactiveness_ = 0.91.

Taken together, these observations show that coaches’ judgments were consistent except for passing speed where alpha coefficient was below 0.7, a value for which the consistency is judged as questionable.

## Discussion

The present study aimed i) to investigate the respective effects of age and soccer practice experience on the CMP in young elite soccer players in a short passing situation, and ii) to compare the objectively determined outcome measures of a new high-technology system (COGNIFOOT) to the more subjective judgements of expert coaches. Based on previous reports [12], we expected substantial improvements in the players’ reactiveness and passing skills from the U12 to the U16 age categories. Our observations went beyond these expectations as we observed linear age-related increases in the CMP. In particular, the passing accuracy, the passing speed and the reactiveness of players improved by 4 centimeters, 2.3 km/h and 30 milliseconds per year, respectively. These age-related CMP changes measured with COGNIFOOT were also observed in expert coaches’ judgments although these were more variable across coaches and age categories. These observations have several implications for talent identification and cognitive-motor training that we discuss below.

### Effect of age on the cognitive-motor performance in soccer

We observed fine changes in CMP performance of elite young players during a short (5 meters) passing situation: in U12 players, the average spatial error was equal to 45 centimeters but decreased by around 4 centimeters per year to reach 30 centimeters at 15 years of age (Fig 2A). Whether purely cognitive or motor factors account for the age-related improvements reported here cannot be answered with our data. The effects of age and expertise level were examined in a battery of non-specific (basic visual function tests) and soccer-specific perceptual-cognitive tests in a population of elite (recruited from English Premier League academies) and sub-elite soccer players aged from 9 to 17 years [27]. In this study, film-based simulations were projected on a large screen and participants responded using pencil and paper. Age, but not expertise, significantly affected the performance of participants in basic visual function tests: the visual acuity and the peripheral awareness improved from 9 to 13 years and from 11 and 15 years of age, respectively. Interestingly, age was a stronger predictor than expertise for two specific tests: a “structured memory recall” test where participants were asked to recall the position of particular players from both teams and an anticipation test where participants had to anticipate the direction of a pass in a 3 *vs* 3 situation. Expertise, but not age, had a significant positive effect on anticipation in 11 *vs* 11 situations and percentage of key players highlighted (the players in a good position to receive the ball in 11 vs 11 situations). In the present study, all the players we tested competed at the elite level: testing novice players at different ages may help in further discriminating between the effect of age and expertise on CMP. While the data clearly indicate that there is a CMP increase with increasing age, we also raised the question whether other parameters such as the ‘accumulated soccer practice’ or the ‘accumulated soccer practice at the elite level’ may even better predict the development of CMP. We observed that *age* is a better predictor followed by *accumulated soccer practice at the elite level* and finally *accumulated soccer practice* for all measured parameters. Using tasks comparable to those tested by Ward and Williams (2003), Roca and Williams [12] reported that the accumulated soccer practice experience during childhood, followed by accumulated soccer practice experience during adolescence were the best predictors of perceptual-cognitive expertise in a population of adult soccer players (around 21 years of age on average) with different levels of expertise (semi-professional, amateur and recreational). The potential separate effects of *accumulated soccer practice at the elite level* and *accumulated soccer practice* were not documented in Roca study. Therefore, including realistic motor responses as in the present study and better discriminating the accumulated soccer practice (elite *vs* non-elite) may help to provide a better understanding of how cognitive and motor components of the CMP evolve with age and soccer practice level.

### Assessing the CMP during short-pass situations in soccer

As the importance of CMP assessment has been identified relatively recently (see [4] for a review), the development of adequate tests is still at its beginning. Concerning soccer passing tests, only few field tests provide quantitative and objective measurement of the passing performance. A widely used test, the Loughborough Soccer Passing Test (LSPT, see [4]), consists in a series of passes executed by players towards soccer-specific, color-coded plastic rebound boards placed along the sides of a 12 m x 9.5 m playfield. A limitation of the LSPT is that the passing accuracy is usually judged by solely one human observer. This can be overcome by measuring passing accuracy using digitizing video footage [28] rather than involving a human observer as in the LSPT studies. In this last study, the passing accuracy and speed during short passing situations (4.2 to 7.9 meters distance) ranged, on average, between 37 and 32 cm and between 42 and 48 km/h in adult recreational *vs* professional players, respectively. The passing accuracy was of the same range as the one observed in our study (30–45 cm across age categories). However, the passing speed was lower in our study (30.7–40.3 km/h across age categories). Interestingly, an increase of 2.3 km/h per year of age (as observed in our regressions) would predict a passing speed equal to 47.2 km/h at the adult age for the U15U16 players we tested, a value comparable to the one observed in adult players [28]. Besides, in these studies [28–31] and in contrast with the present study, the height of the target was not manipulated and all passes were performed at floor level. This could have had an effect on the measured parameters as we observed statistically significant effects of the *target lateral position x vertical position* and *vertical target position* on the passing accuracy and response times, respectively.

While these different testing protocols proved to successfully discriminate between the performance levels of male, female and adolescent players at the motor execution level [28–31], the visual environment was always static (four rebound boards/targets located at fixed locations) and visual targets are visible for the whole test duration. Thus, the cognitive aspect of the test can be rated as not very demanding and cannot be easily adapted. This means that no measurements of the response times (not documented in the mentioned studies) or passing speed in more dynamic environments, i.e. with targets appearing for random durations and at random positions, in presence of various numbers of visual distractors, as observed during real competition, can be provided. For this purpose, the COGNIFOOT system was developed that provides quantitative measurements automatically and in real-time. In the present study, we designed a test with higher perceptual demands and highly objective quantification of the outcome measures in order to judge CMP in young soccer players. Although we simulated simple visual stimuli, we could observe a statistically significant effect on the passing accuracy: the passing accuracy was greatest for targets at floor level, without visual distractors and for stimulus durations longer than 200 ms. It should be noted that new systems like COGNIFOOT allow a fine simulation of a theoretical infinite number of situations and the testing of several components of perceptual skills while at the same time recording accurately-measured CMP variables, a huge difference to the field tests developed so far.

### Comparison between Cognifoot measures and coaches’ evaluation

Although more and more complex soccer tests and test batteries are developed, an influential part in talent selection is still the judgement of the coaches and/or talent scouts. Therefore, we compared the results obtained with COGNIFOOT with the evaluation provided by well-educated and well-experienced coaches. Importantly, we obtained the ratings of the coaches in two different ways by asking them to provide *absolute* and *relative* judgments: *absolute* judgments were evaluations of coaches without providing a specific reference while *relative* judgments asked coaches to give their judgements with reference to one well-known player–the best player of the oldest category. We hypothesized that providing a reference would enable coaches to better differentiate their judgements across age groups and avoid biases due to taking into account the potential of young players instead of their actual CMP-performance. In line with our hypothesis, the mean *relative* coaching scores were closer to the objectively-measured scores by the COGNIFOOT (Figs 5B, 6B and 7B) and reflected better the progression with age and/or experience. This was also indicated by smaller variability of the data when using the relative compared to the absolute coaches’ judgement (Table 1). However, irrespective of the way coaches judged the performance of their players (absolute or relative judgement) it has to be emphasized that only the mean score across all coaches for each individual player led to considerably reduced data dispersion and increased correlation coefficients. For the relative judgements, this procedure resulted in comparable regression line parameters as the ones obtained with COGNIFOOT (Fig 5B). This questions the reliability of evaluations that are based on one coach/scout only–especially if they are made without a specific reference, which may be important for talent selection procedures.

### Implications for talent selection

The comparison of absolute and relative judgements in the present study has indicated that clear reference values, either the level of one specific player or a certain performance criterion may help to reduce variability across coaches’ judgements. This may be an important aspect to improve talent identification programs. However, in any case, objective measurements of quantitative data like those provided by the COGNIFOOT system have an even greater discriminative power as it relies on physical measurements of clearly-defined CMP parameters. Furthermore, as only the mean of the coaches’ score was comparable to the COGNIFOOT score, talent selection based on the judgement of one single coach may inherit serious limitations as mentioned earlier. At the same time, it has to be noted that the COGNIFOOT system only assessed CMP in short pass situations and does therefore not allow a more global assessment. Therefore, future approaches using high-technology systems should be extended by including more realistically simulated situations that may further strengthen the ecological validity of these systems. In particular, the challenge would be to extend CMP measurements to soccer-specific CMP-skills such as long passes, free kicks, penalty or corner kicks as well as collective actions leading to one of these actions. Such progress may allow more reliable talent selection processes in the future.

### Cognitive-motor training in soccer

The efficiency of any training program can only be assessed by having pre-training and post-training measurements. For physiological assessments in soccer, reliable field tests like the Yo-Yo test [32] are widely used in elite soccer. The COGNIFOOT prototype used in this study offers an interesting possibility for providing such objective and quantitative measurements of the CMP. In our study, we noticed few interaction effects between motor factors (linked to the difficulty to hit laterally-high targets) and visual factors (stimulus duration and number of visual distractors) on the passing accuracy (see Figure A of S1 File) or on the response times (see Figure C of S1 File). Adding a textured background or a real image (or movie) may more significantly affect passing accuracy and response times. Exposing players to a variety of specific and non-specific stimuli may provide new insights into the interaction between the cognitive demands of a situation and the quality of motor execution. It would also be interesting to reproduce these tests in combination with fatigue protocols in order to quantify a potential fatigue-effect on the CMP of soccer players (see for example [33] and [34].For example, shots and passes accuracy and/or speed were found to significantly decrease during the second half compared to the first half of a soccer match simulation [34]. However, despite accurate and reliable measurements of the CMP, current limits of this prototype should be overcome in the future: i) the ball was static at the beginning of each passing sequence and ii) the stimuli were static. Besides, future versions of the COGNIFOOT system may integrate ball launching devices and different types of visual environments (with static or moving targets, or freely-chosen pass destination when facing real game situations) in order to automatize the sequence of passes and for enhanced ecological validity. This would allow using the COGNIFOOT for testing different CMP training protocols.

## Conclusions

Measuring the CMP of soccer players in realistic situations is of particular interest. Here we report objective CMP measurements showing linear improvements of the cognitive-motor performance with age in short-pass situations in young elite soccer players that, to the best of our knowledge, are documented for the first time. These observations confirmed that the pre-adolescence–mid-adolescence period is of critical developmental stage for the acquisition of superior perceptual-cognitive skills in soccer [12]. Besides, objectively-measured scores were comparable to mean expert coaches’ scores (although these were more variable across coaches and age categories), showing that high-technology systems like COGNIFOOT can be useful for talents’ identification. Through its high adaptability and real-time properties, such systems also offer a great potential for cognitive-motor training. Future studies using comparable methodologies are required to further document how cognitive and motor skills of soccer players evolve with soccer practice in adult players and with other factors like fatigue or external environmental features (e.g. humidity or temperature).

## Supporting information

S1 File Cognifoot measurements across all tested conditions (number of visual distractors, stimulus duration and target positions) and individual coaches’ judgments. Figure A: Passing spatial error of elite young soccer players. Figure B: Passing speed of elite young soccer players. Figure C: Response times of elite young soccer players. Figure D: Passing accuracy scores measured by the Cognifoot system or judged by coaches. Figure E: Passing speed scores measured by the Cognifoot system or judged by coaches. Figure F: Reactiveness scores measured by the Cognifoot system or judged by coaches.
(DOCX)
