# Supplementary material for: Effects of age on the soccer-specific cognitive-motor performance of elite young soccer players: Comparison between objective measurements and coaches’ evaluation
Source: PLoS One. 2017 Sep 27;12(9):e0185460. doi: 10.1371/journal.pone.0185460 (PMC5617197; doi:10.1371/journal.pone.0185460)
Supplement: S1 File — Figure A: Passing spatial error of elite young soccer players. Figure B: Passing speed of elite young soccer players. Figure C: Response times of elite young soccer players. Figure D: Passing accuracy scores measured by the Cognifoot system or judged by coaches. Figure E: Passing speed scores measured by the Cognifoot system or judged by coaches. Figure F: Reactiveness scores measured by the Cognifoot system or judged by coaches. (DOCX) [file pone.0185460.s001.docx]

**Effects of age on the soccer-specific cognitive-motor performance of elite young soccer players: comparison between objective measurements and coaches’ evaluation**

**Supplementary Material**

**Results**

**Cognifoot measurements**

**Passing accuracy**

The linear regression analysis applied on all recorded passes confirmed the effect of age (F_(1, 43)_=88.1, p<0.001). The target lateral position and vertical positions (panels A and B in Fig A, respectively), the number of visual distractors (panel C in Fig A) and the duration of the stimuli (panel D in Fig A) did not significantly affect the passing spatial error (p>0.05). However, two types of statistically significant interaction effects were observed. First, a statistically significant “*target lateral position x vertical position*” effect (F_(4, 172)_=4,66, p<0.01) on the passing spatial error was observed. *Post-hoc pairwise comparisons (Bonferroni test) revealed that passes towards the FLOOR-LEFT, FLOOR-CENTER and FLOOR-RIGHT positions (30.9 ± 4.3 cm, 29.3 ± 3.8 cm and 31.7 ± 3.4 cm, respectively) were significantly more accurate than any other remaining target position (for which passing accuracy errors ranged between 35.7 and 43.4 cm).* In addition, a “*target lateral position x vertical position x age*” interaction effect (F_(4, 172)_=4,38, p<0.01) revealed that this higher accuracy for screen center/floor level passes was less pronounced with increasing age. Second, “*target vertical position x number of visual distractors x stimulus duration*” and “*target vertical position x number of visual distractors x stimulus duration x age*” interaction effects were observed (F_(12, 516)_=2,0, p=0.04 and F_(12, 516)_=1,83, p=0.04, respectively). This reveals that the passing spatial error was smaller for targets at floor level, without visual distractors and for stimulus durations longer than 200 ms. We observed a decreasing dispersion of the data points with increasing age (panels A, B, C and D in Fig A).


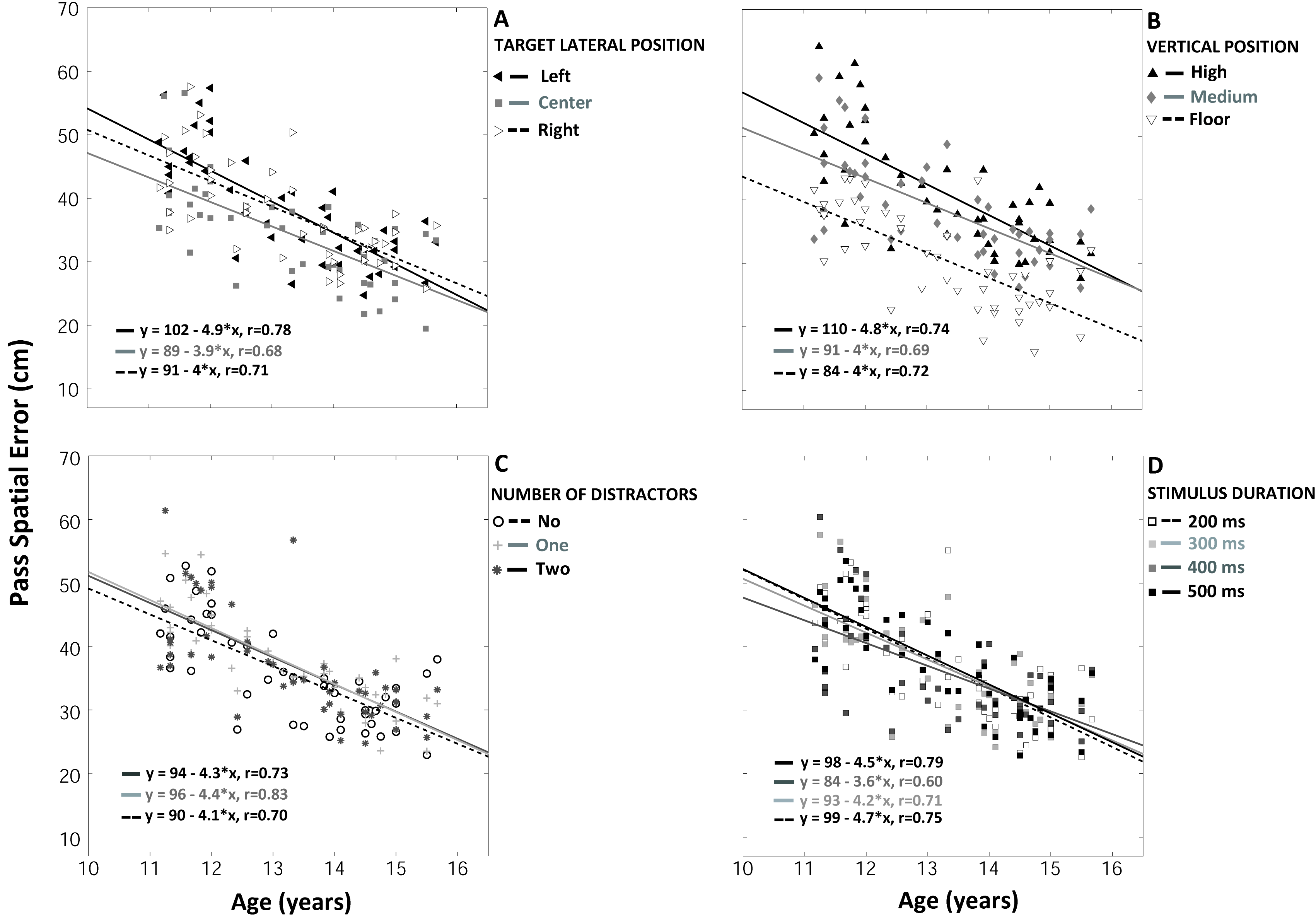


**Fig A:** **Passing spatial error of elite young soccer players.** Passing spatial error as a function of age and target lateral position (A), vertical position (B), number of visual distractors (C) and stimulus duration (D). Note that significant target lateral position x vertical position (passes were more accurate with age and for targets located at floor level) and vertical position x number of distractors x stimulus duration interaction effect were observed (see text for details).

**Passing speed**

The linear regression analysis applied on all recorded passes confirmed the main effect of age (F_(1, 43)_=27.6, p<0.001) on the passing speed (Fig B). However, no statistically significant effect of the target lateral position and vertical position (panels A and B in Fig B, respectively), the number of visual distractors (panel C in Fig B) and the duration of the stimuli (panel D in Fig B) on the passing speed was observed (p>0.05, no interaction effect). We also observed a decreasing dispersion of the data points with increasing age (panels A, B, C and D in Fig B ).

**
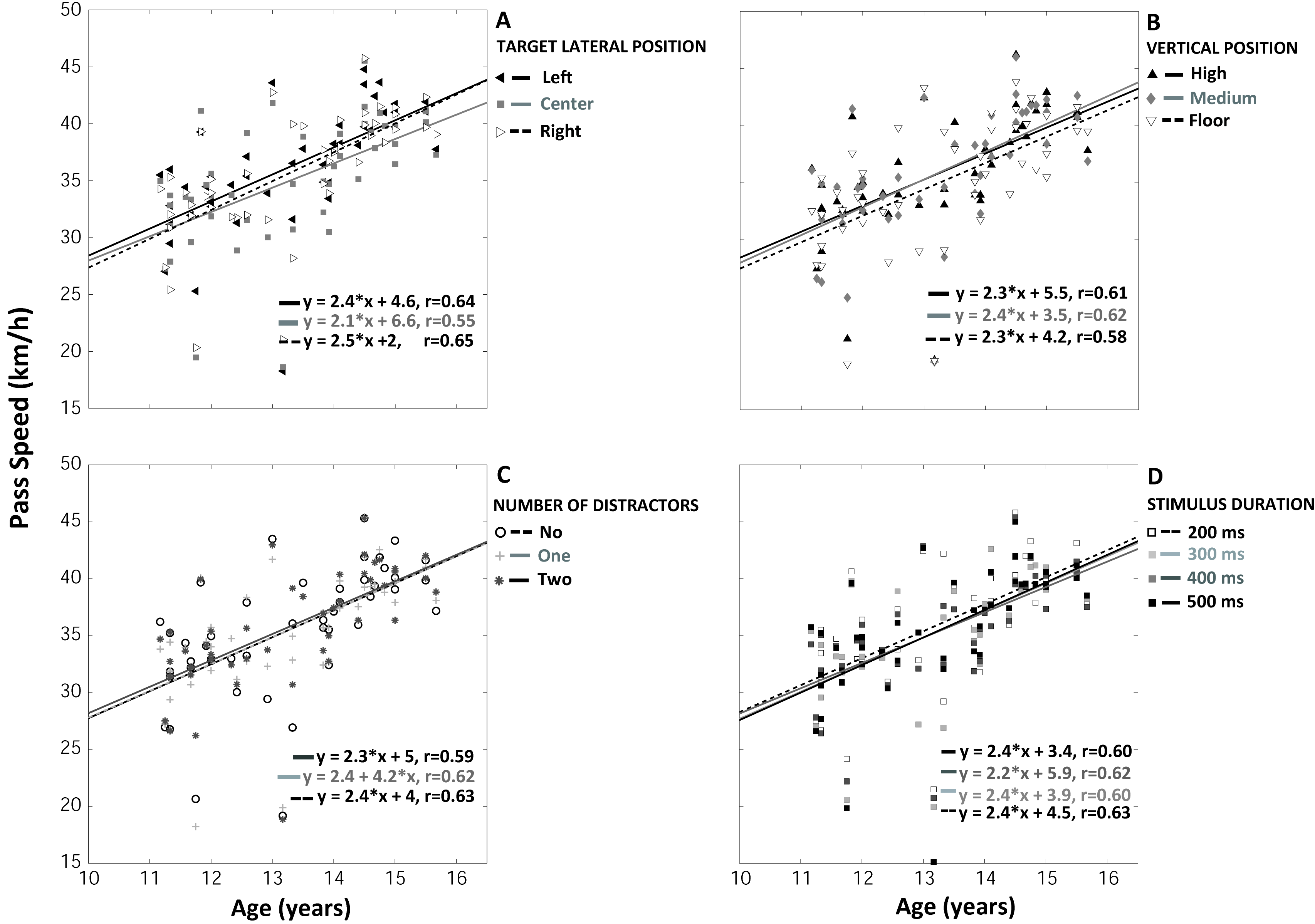
**

**Fig B:** **Passing speed of elite young soccer players.** Passing speed as a function of age and target lateral position (A), vertical position (B), number of visual distractors (C) and stimulus duration (D). No statistically significant effect of these conditions on the passing speed/age relationship was observed.

**Response time**

The linear regression analysis applied on all recorded passes confirmed the main effect of age on the response time although the effect was just at significance level (F_(1, 43)_=4.01, p=0.051, panels A, B, C and D in Fig C). We observed a statistically significant effect of the vertical target position (F_(2, 86)_=4.14, p=0.02) and a “*vertical target position x age*” interaction effect (F_(2, 86)_=5.7, p<0.01) on the response times. Response times were shorter for targets located at floor level and decreased with increasing age (panel B in Fig C). No other significant effects were observed.

**
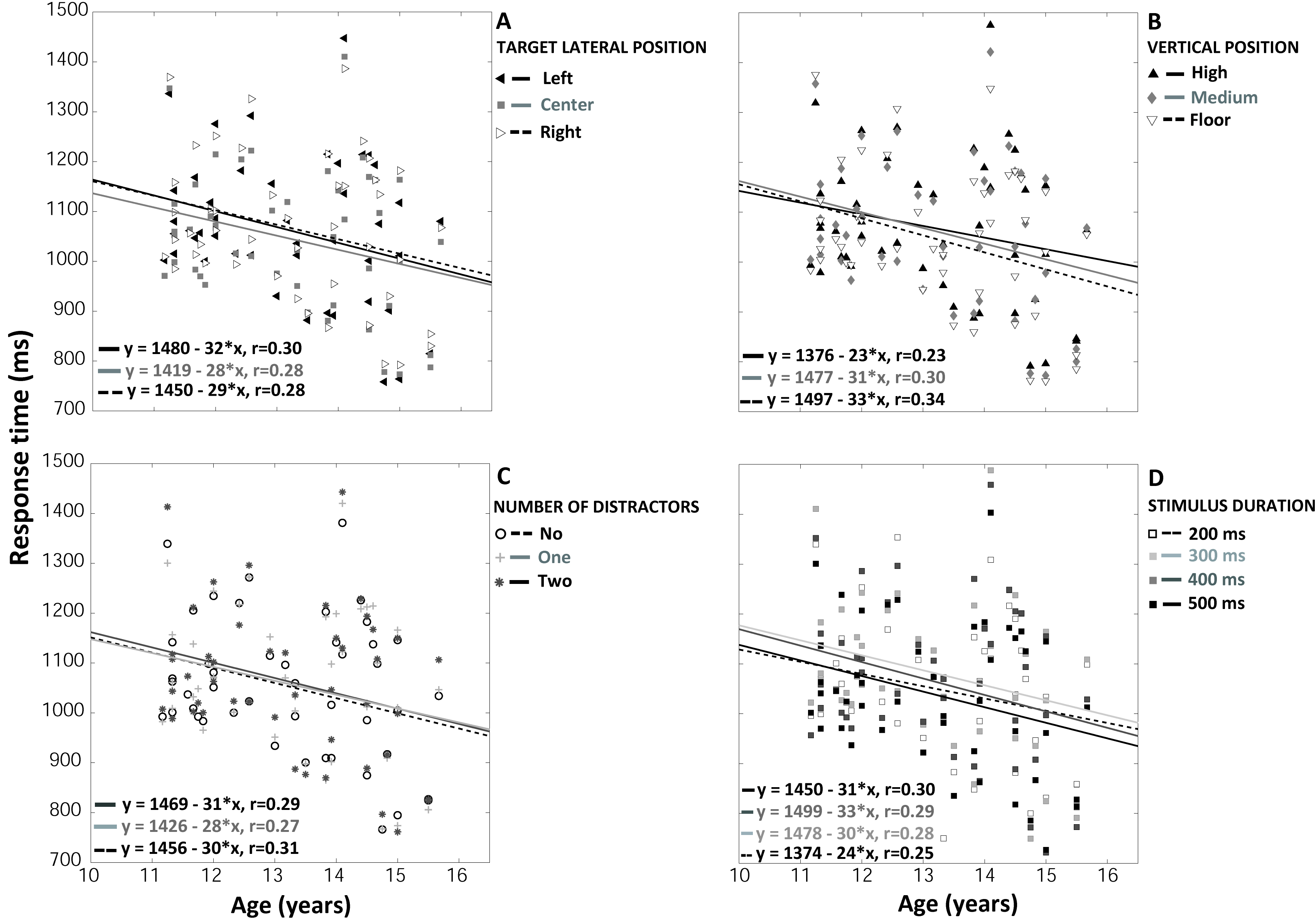
**

**Fig C:** **Response times of elite young soccer players.** Response times as a function of age and target lateral position (A), vertical position (B), number of visual distractors (C) and stimulus duration (D). Note that a significant target vertical position x age (response times were shorter with increasing age and for targets located at floor level) interaction effect was observed (see text for details).

**Coaches’ judgements vs Cognifoot measurements**

The players’ passing accuracy/speed and reactiveness detailed in the previous section were transformed into 5 point-scale scores and compared to the expert coaches’ judgments (see *Methods* of the main manuscript). As the linear relationship between COGNIFOOT scores and age were highlighted in the previous paragraphs, the focus here is more on the scores provided by coaches in order to a) determine the extent to which they were also affected by the age of the players and b) to test how close they were to the objective measurements provided by the COGNIFOOT system.

**Passing accuracy judgments**

The PA_score_ measured by the COGNIFOOT system linearly increased with age (F_(1, 44)_=91.8, p<0.001, r=0.82, panel A in Fig D). The individual *absolute* PA_scores_ (N=201) provided by coaches also linearly increased with age (F_(1, 199)_=13.3, r=0.25, p<0.01, panel B in Fig D). The same linear relationship was observed for individual *relative* PA_scores_ (F_(1, 196)_=36.4, , r=0.40 p<0.01, panel C in Fig D- N=198, 201 *minus* 2 missing judgments *minus* the judgment of the “reference” player who was used to provide relative judgments). Both *absolute* and *relative* coaches’ judgments were marked by a higher dispersion of data points compared to COGNIFOOT scores, resulting in weaker correlation coefficients. The slope of the *relative* coaches’ scores/age regression line (panel C in Fig D) was close to the one of the COGNIFOOT. The higher data dispersion in coaches’ judgments emerged from inter-coaches differences in judging the score of a same player.


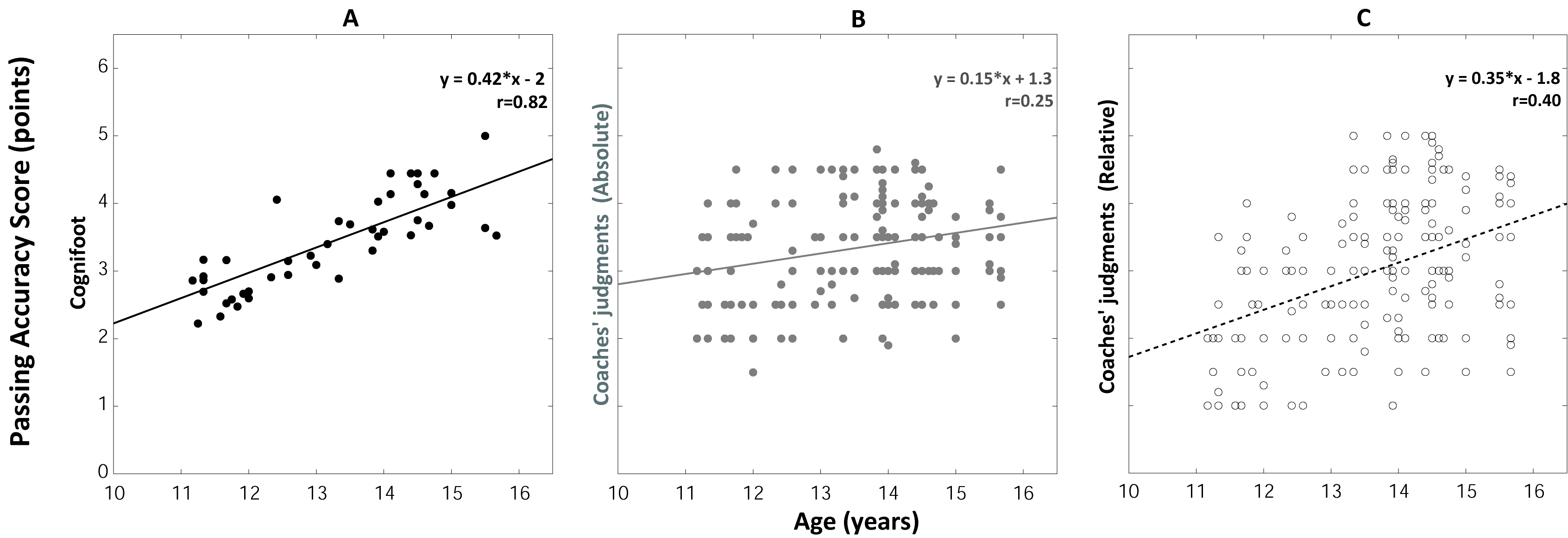


**Fig D:** **Passing accuracy scores measured by the Cognifoot system or judged by coaches.** Passing accuracy scores (5 point-scale) *vs* Age as measured by the Cognifoot system (A), or as judged by coaches (individual coach absolute -B- and relative -C- judgments, see text for details).

**Passing speed judgments**

The PS_score_ measured by the COGNIFOOT system linearly increased with age (F_(1, 44)_=28.6, p<0.001, r=0.63, panel A in Fig E ). The individual *absolute* PS_scores_ (N=201) provided by coaches also linearly increased with age (F_(1, 199)_=18.9, p<0.001, r=0.29, panel B in Fig E). The same linear relationship was observed for individual *relative* PS_scores_ (F_(1, 196)_=42.8, r=0.42, p<0.001, panel C in Fig E). Both *absolute* and *relative* coaches’ judgments are marked by a higher dispersion of data points compared to COGNIFOOT scores, resulting in weaker correlation coefficients.


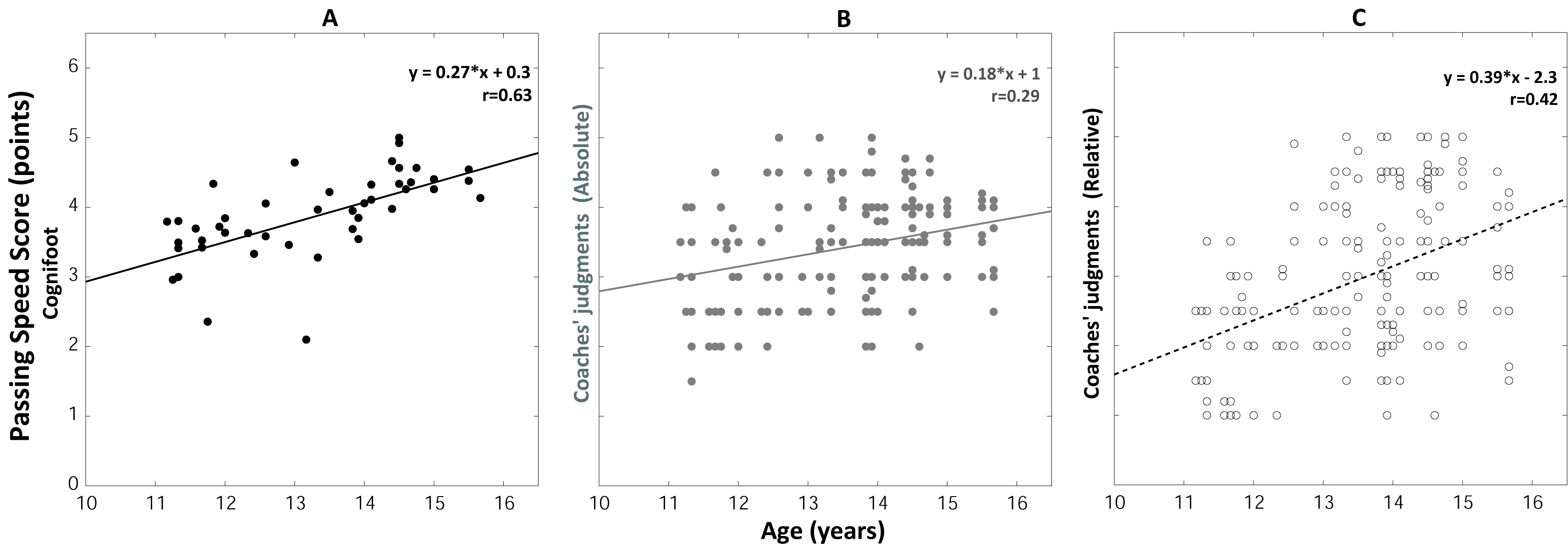


**Fig E:** **Passing speed scores measured by the Cognifoot system or judged by coaches.** Passing speed scores (5 point-scale) *vs* Age as measured by the Cognifoot system (A), or as judged by coaches (individual coach absolute -B- and relative -C- judgments, see text for details).

**Reactiveness judgments**

The RE_score_ measured by the COGNIFOOT system linearly increased with age (F_(1, 44)_=4.7, r=0.31, p=0.036, panel A in Fig F). The individual *absolute* RE_scores_ (N=201) provided by coaches also linearly increased with age (F_(1, 199)_=11.1, r=0.23, p<0.01, panel B in Fig F). The same linear relationship was observed for individual *relative* RE_scores_ (F_(1, 196)_=35.3, r=0.39, p<0.001, panel C in Fig F). It is remarkable that weak correlation coefficients between RE_score_ and age are observed both for COGNIFOOT and coaches’ data. This can be explained by low regression line slopes (equal to 0.11, panel A in Fig F) in the COGNIFOOT data (compared to the one of PA and PS scores) and by high data dispersion in the coaches’ scores, respectively.

**
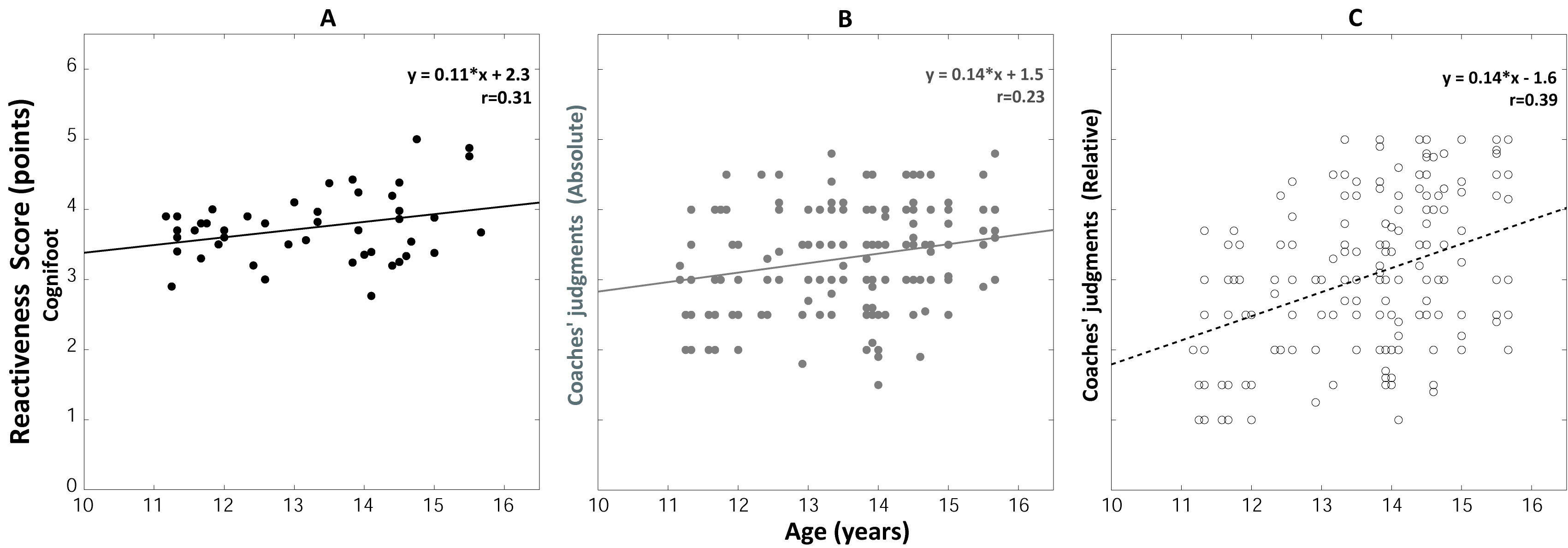
**

**Fig F:** **Reactiveness scores measured by the Cognifoot system or judged by coaches.** Reactiveness scores (5 point-scale) *vs* Age as measured by the Cognifoot system (A), or as judged by coaches (individual coach absolute -B- and relative -C- judgments, see text for details).
